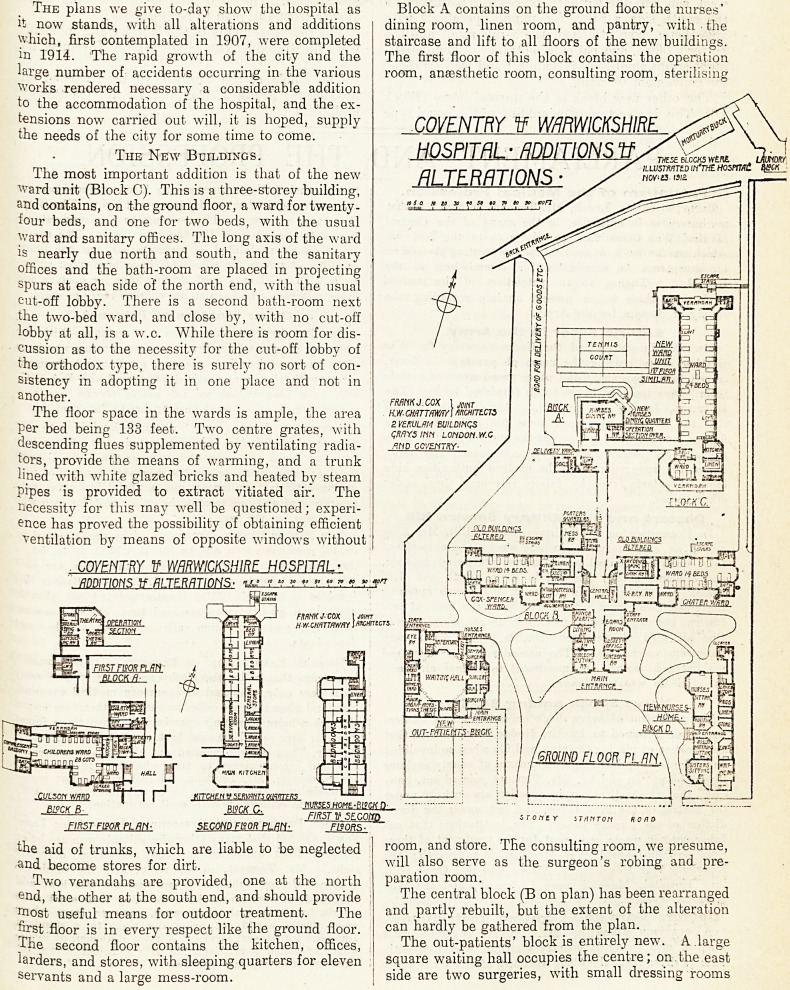# Coventry and Warwickshire Hospital

**Published:** 1916-01-29

**Authors:** 


					January 29, 1916. THE HOSPITAL 393
HOSPITAL ARCHITECTURE AND CONSTRUCTION.
Coventry and Warwickshire Hospital.
The plans we give to-day show the hospital as
it now stands, with all alterations and additions
which, first contemplated in 1907, were completed
in 1914. The rapid growth of the city and the
large number of accidents occurring in the various
Works rendered necessary a considerable addition
to the accommodation of the hospital, and the ex-
tensions now carried out will, it is hoped, supply
the needs of the city for some time to come.
The New Buildings.
The most important addition is that of the new
Ward unit (Block C). This is a three-storey building,
and contains, on the ground floor, a ward for twenty-
four beds, and one for two beds, with the usual
Ward and sanitary offices. The long axis of the ward
is nearly due north and south, and the sanitary
offices and the bath-room are placed in projecting
spurs at each side of the north end, with the usual
cut-off lobby. There is a second bath-room next
the two-bed ward, and close by, with no cut-off
lobby at all, is a w.c. While there is room for dis-
cussion as to the necessity for the cut-off lobby of
the orthodox type, there is surely no sort of con-
sistency in adopting it in one place and not in
another.
The floor space in the wards is ample, the area
per bed being 133 feet. Two centre grates, with
descending flues supplemented by ventilating radia-
tors, provide the means of warming, and a trunk
lined with white glazed bricks and heated by steam
pipes is provided to extract vitiated air. The
necessity for this may well be questioned; experi-
ence has proved the possibility of obtaining efficient
ventilation by means of opposite windows without
the aid of trunks, which are liable to be neglected
and become stores for dirt.
Two verandahs are provided, one at the north
end, the other at the south end, and should provide
niost useful means for outdoor treatment. The
first floor is in every respect like the ground floor.
The second floor contains the kitchen, offices,
larders, and stores, with sleeping quarters for eleven
servants and a large mess-x-oom.
Block A contains on the ground floor the nurses'
dining room, linen room, and pantry, with the
staircase and lift to all floors of the new buildings.
The first floor of this block contains the operation
room, anaesthetic room, consulting room, sterilising
room, and store. TRe consulting room, we presume,
will also serve as the surgeon's robing and pre-
paration room.
The central block (B on plan) has been rearranged
and partly rebuilt, but the extent of the alteration
can hardly be gathered from the plan.
The out-patients' block is entirely new. A .large
square waiting hall occupies the centre; on.the east
side are two surgeries, with small dressing rooms
The plans we give to-day show the hospital as Block A contains on the ground floor the nurses'
it now stands, with all alterations and additions dining room, linen room, and pantry, with ? the
which, first contemplated in 1907, were completed staircase and lift to all floors of the new buildings,
in 1914. The rapid growth of the city and the The first floor of this block contains the operation
large number of accidents occurring in the various room, anaesthetic room, consulting room, sterilising
Works rendered necessary a considerable addition
to the accommodation of the hospital, and the ex-
tensions now carried out will, it is hoped, supply COVENTRY V WARWICKSHIRE.
tne needs of the city tor some time to come.
The New Buildings.
HOSPITAL ? ADDITIONS If
The most important addition is that of the new Rl~TE.RRTlQNS
Ward unit (Block 0). This is a three-storey building,
and contains, on the ground floor, a ward for twenty-
four beds, and one for two beds, with the usual
Ward and sanitary offices. The long axis of the ward
is nearly due north and south, and the sanitary
offices and the bath-room are placed in projecting
spurs at each side of the north end, with the usual
cut-off lobby. There is a second bath-room next
the two-bed ward, and close by, with no cut-off
lobby at all, is a w.c. While there is room for dis-
cussion as to the necessity for the cut-off lobby of
the orthodox type, there is surely no sort of con-
sistency in adopting it in one place and not in
another.
The floor space in the wards is ample, the area
per bed being 133 feet. Two centre grates, with
descending flues supplemented by ventilating radia-
tors, provide the means of warming, and a trunk
lined with white glazed bricks and heated by steam
pipes is provided to extract vitiated air. The
necessity for this may well be questioned; experi-
ence has proved the possibility of obtaining efficient
ventilation by means of opposite windows without
. COVFNTRY 1 W3BEICKSH1RL HOSPITAL-
. miTIOtiS V ALTERATIONS^
~BL?CK B' HMJi. F/R5T v 5EQ0m?
FIRST F120R PL RH- SECOND FIS OR PLAN- FISORS^
the aid of trunks, which are liable to be neglected
and become stores for dirt.
Two verandahs are provided, one at the north
end, the other at the south end, and should provide
most useful means for outdoor treatment. The
first floor is in every respect like the ground floor.
The second floor contains the kitchen, offices,
larders, and stores, with sleeping quarters for eleven
servants and a large mess-room.
room, and store. The consulting room, we presume,
will also serve as the surgeon's robing and pre-
paration room.
The central block (B on plan) has been rearranged
and partly rebuilt, but the extent of the alteration
can hardly be gathered from the plan.
The out-patients' block is entirely new. A large
square waiting hall occupies the centre; on.the east
side are two surgeries, with small dressing rooms
394 THE HOSPITAL January 29, 1916.
attached, and a room for the dental surgeon; on
the north side is the dispensary and patients' exit,
and a room for the ophthalmic surgeon. On the
west side are the sanitary offices for patients; on
the south side a room for minor operations with
anaesthetic room attached, the ?-ray room and the
porter's office, and patients' entrance. The depart-
ment seems small, but is compact and well arranged
for convenient working.
The other new block is the nurses' home (Block
D). This is a rectangular building of the usual type
and three floors high. The sitting rooms are all on
the ground floor, and comprise one for sisters, one
for nurses, a waiting room, and a room for assist-
ant matron. The total number of bedrooms would
appear from the plan to be thirty-six.
The rearrangements of the laundry and mortuary
were illustrated in The Hospital on November 23,
1912.
The whole of the works have been carried out
by Messrs. Frank J. Cox and H. W. Chattaway,
architects.

				

## Figures and Tables

**Figure f1:**